# Editorial: Everything edamame: Biology, production, nutrition, sensory and economics

**DOI:** 10.3389/fpls.2022.976008

**Published:** 2022-07-27

**Authors:** Martin M. Williams, Bo Zhang, Xujun Fu, Jeremy Ross

**Affiliations:** ^1^Global Change and Photosynthesis Research Unit, USDA-Agricultural Research Service, Urbana, IL, United States; ^2^School of Plant and Environmental Sciences, Virginia Polytechnic Institute and State University, Blacksburg, VA, United States; ^3^Institute of Crop and Nuclear Technology Utilization, Zhejiang Academy of Agricultural Sciences, Hangzhou, China; ^4^Crop, Soil, and Environmental Sciences, University of Arkansas System Division of Agriculture, Little Rock, AR, United States

**Keywords:** beer bean, green soybean, immature soybean seed, mukimame, vegetable soybean

Globally, demand for edamame, also called vegetable soybean, is on the rise. As a crop grown and consumed in East Asian countries for centuries, in recent decades edamame has been consumed with a rising trend in new parts of the world, including the U.S. Edamame is recognized as a healthy plant-based protein which is also rich in vitamins, dietary fiber, and isoflavones. Most commonly, edamame soybean is consumed as a snack or added to salads, soups, stews, or dips.

In some countries, such as China and Taiwan, a well-developed edamame industry exists. In other countries, such as the U.S., edamame remains a crop in its infancy. In between the small, niche market and the large national market, there exists a void where the market is poorly defined and hurdles to growing an industry are both varied and numerous. Regardless of the maturity of the edamame value chain in a particular country, addressing consumer expectations and improving the sustainability of food production in the face of global change requires innovation.

This Research Topic aims to provide the latest achievements of edamame research, in multiple disciplines, to identify the status of the edamame value chain from field to fork. We are honored to receive submissions of many manuscripts on edamame. After a vigorous review and revision procedure, 12 articles were collected in this Research Topic, covering consumer preferences, crop physiology and production, economics, marketing, food processing, and plant breeding.

Despite the U.S. being a leading producer of grain-type soybean, multiple attempts over the last century to develop a substantial domestically produced edamame industry has gained minimal traction. Production of specialty crops such as edamame are generally high-risk, high-reward endeavors. In this Research Topic, Neill and Morgan conduct a risk assessment of U.S. edamame, accounting for production, finance, regulatory, price, and human resource risks. They note that numerous assumptions made about edamame production are due to lack of data. While the authors conclude edamame production in the U.S. has great potential, the authors call for private-public partnerships to shed light on potential risks and facilitate the realization of domestic production.

Growing demand for edamame in the U.S. presents a unique challenge because the food products may be minimally familiar to consumers. A greater understanding of U.S. consumer preferences for edamame was a major thrust in this Research Topic. Carneiro, Adie et al. investigated the role of overall appearance and color characteristics in consumer's acceptability of edamame beans. Using beans from 10 edamame genotypes grown in Virginia, the authors used a sensory panel to compare “dark” vs. “light” green edamame beans. They found consumers favored dark green edamame beans. Lord et al. evaluated consumer preferences for edamame marketed as fresh, local, organic, or beans on-the-stalk. They found consumers were willing to pay more for fresh, local, and organic edamame, while they were willing to pay less for beans on-the-stalk. Carneiro, Drape et al. investigated consumers' preferences and intentions to buy edamame grown in the U.S. They found domestically grown, in-shell edamame products were preferred compared to shelled edamame or imported products. In addition, survey respondents exhibited higher intention to buy fresh edamame relative to frozen edamame. Unfortunately, information on product characteristics desirable to consumers is often obtained in the late stages of edamame variety development due to the cost and complexity of traditional sensory methodologies. The review paper by Carneiro, Duncan, O'Keefe, Yin et al. argues the importance of integrating sensory attributes of edamame with germplasm improvement. They call for the development of alternative sensory methods that are simple, fast, and effective to obtain consumer data. Indeed, one paper in the Research Topic directly linked sensory evaluations with edamame breeding. Carneiro, Duncan, O'Keefe, Yu et al. used a sensory evaluation to identify edamame genotypes and sensory attributes preferred by consumers to support breeding selection criteria. They found traits described as “bitter,” “sour,” or “starchy” appeared less acceptable, while “salty” and “sweet” appeared more acceptable.

The need for improved edamame cultivars through plant breeding was the subject of three additional papers in this Research Topic. Yuan et al. evaluated the volatile compounds of 30 edamame core cultivars from a breeding program in Hangzhou, China. They found that the composition and concentration of volatile compounds from the cultivars examined varied with cultivar ecotype, objectives of the breeding selection, and geographic origin. They conclude volatile fingerprints of lines in a breeding program can be used to improve the desirable aroma of future cultivars. Sensory attributes are not the only traits important in edamame breeding. Effectiveness of mechanical edamame harvest could be improved with an understanding of plant architectural traits. Dhakal et al. used digital imaging technology and computer vision algorithms to characterize plant architecture and identify genetic control of these traits. They found a combination of multiple topological features that contribute to the overall pod numbers on a plant. They also identified potential candidate genes associated with pod number. Finally, Kao et al. describe a core collection of edamame accessions in Taiwan, which has created a successful edamame industry in the last half-century. The authors developed an algorithm to select 30 accessions with maximum pairwise genetic distance from a collection of 200 unique accessions. They conclude the core collection will benefit future research and breeding efforts since it retains diversity and genetic variability of edamame suitable for Taiwan.

Potassium is essential to plant growth and potassium efficiency has been the study of previous research in edamame. Liu et al. examine root potassium affinity-associated drivers and photosynthesis in edamame with different potassium efficiency. They conclude that stronger root potassium affinity drivers associated with photosynthetic adaptability to low potassium stress were key factors in determining the potassium high efficiency of edamame.

Even with an edamame cultivar capable of delivering sensory traits important to the consumer, determining the optimal time to harvest the crop is currently a combination of art and science. The size, color, and uniformity of both seeds and pods, as well as protein, oil, and starch components within the seed, change dynamically during reproductive plant growth. Moseley, Paulo da Silva et al. developed an Edamame Harvest Quality Index and demonstrate its responsiveness to planting date and cultivar. They conclude the research will help define a planting and harvesting strategy for edamame production in the U.S. Mid-South.

The current market for edamame consists of either fresh, frozen, roasted, or freeze-dried products; however, producing a high moisture content product that is shelf stable necessitates an improved pasteurization technique. Moseley, Mozzoni et al. compared acid-treatment to boiling on edamame texture and color as well as the effect of additions and cultivars on acid-treatment. They found the acid-treatment, including the addition of turmeric, helped maintain quality of canned edamame seeds. This work identifies a promising path to new edamame products not currently available to many consumers.

Collectively, the scope of papers on this Research Topic illustrates a diversity of research efforts on improving the edamame value chain from field to fork. The collection offers new insights for countries where edamame is mainstream, such as China and Taiwan, as well as places such as the U.S. where the market is growing and domestic production appears viable.

## Author contributions

MW drafted the manuscript. BZ, XF, and JR reviewed and edited the manuscript. All authors contributed to the article and approved the submitted version.

## Conflict of interest

The authors declare that the research was conducted in the absence of any commercial or financial relationships that could be construed as a potential conflict of interest.

## Publisher's note

All claims expressed in this article are solely those of the authors and do not necessarily represent those of their affiliated organizations, or those of the publisher, the editors and the reviewers. Any product that may be evaluated in this article, or claim that may be made by its manufacturer, is not guaranteed or endorsed by the publisher.

## Author disclaimer

Any opinions, findings, conclusions, or recommendations expressed in this publication are those of the authors and do not necessarily reflect the view of the U.S. Department of Agriculture. Mention of trade names or commercial products in this publication is solely for the purpose of providing specific information and does not imply recommendation or endorsement by the U.S. Department of Agriculture. USDA is an equal opportunity provider and employer.

